# Comparison of Hand-Wrist Findings of Rheumatoid Arthritis Patients According to the Use of Synthetic and Biological Disease-Modifying Antirheumatic Drugs (DMARDs): A Clinical, Radiographic, and Ultrasonographic Study

**DOI:** 10.7759/cureus.46876

**Published:** 2023-10-11

**Authors:** Cigdem Cinar, Yunus Emre Dogan, Halil Harman, Zeynep Yurttutmus, Nazire Bagatir, Muhsin Doran, Kadriye Öneş

**Affiliations:** 1 Department of Interventional Physiatry, Biruni University, Istanbul, TUR; 2 Department of Physiotherapy, Istanbul Physical Therapy and Rehabilitation Health Application and Research Center, Istanbul, TUR; 3 Department of Rheumatology, Kanuni Sultan Suleyman Health Application and Research Center, Istanbul, TUR; 4 Department of Physiotherapy, Yalvac Public Hospital, Istanbul, TUR; 5 Department of Physiotherapy, Istanbul Physical Therapy and Rehabilitation Health Application and Research Center, İstanbul, TUR

**Keywords:** wrist, ultrasonography, rheumatoid arthritis, hand, dmards

## Abstract

Objective: The aim of this study is to evaluate the hand-wrist findings in patients with rheumatoid arthritis (RA) using synthetic and a combination of synthetic and biological disease-modifying antirheumatic drugs (DMARDs) in terms of ultrasonographic, clinical, and radiographic data.

Methods: The study is designed as a cross-sectional study, and 31 RA patients followed up in the rheumatology outpatient clinic were enrolled. Nineteen patients were using only synthetic DMARDs, and 12 patients were using a combination of synthetic and biological DMARDs. The clinical data of each patient were recorded simultaneously. Disease Activity Score-28 (DAS-28) was used for the assessment of disease activation, and the Health Assessment Questionnaire (HAQ) score was used for the evaluation of general health status. Bilateral proximal interphalangeal (PIP), metacarpophalangeal (MCP) joints, and the radiocarpal, ulnocarpal, and midcarpal joints of the patients were examined by ultrasonography (US). The Sharp-van der Heijde modified score was used to determine the radiographic damage.

Results: There was no significant difference between the two groups in terms of demographic data, clinical findings, ESR, and CRP. When the groups were compared in terms of right and left PIP, MCP, and radiocarpal, ulnocarpal, and midcarpal synovitis grade total scores, no significant difference was found between the two groups. Radiographic total joint space scores were significantly lower in the group receiving only synthetic DMARD treatment (p=0.047) and 25-OH vitamin D levels were significantly higher (p=0.008).

Conclusion: This study revealed that there was no significant difference between groups except radiographic total joint space scores.

## Introduction

Rheumatoid arthritis (RA) is an autoimmune inflammatory disease characterized by progressive symmetrical polyarthritis. Intra-articular and peri-articular structures are destroyed as a result of the inflammation of the synovial membrane. In patients receiving inadequate treatment, RA may lead to disability [1]. The global prevalence of RA has been reported as approximately 0.5% [2].

Detecting the disease activity in RA is essential for the determination of both treatment efficacy and prognosis. Thus, a complete clinical examination and sensitive imaging methods are important in the follow-up of the disease process. The main goal in current RA treatment is clinical remission. However, various studies have shown that subclinical inflammation detected by ultrasonography (US) and MRI persists. They reported that radiographic damage progresses despite the clinical remission [3,4].

There are studies that mentioned that US and MRI may be used in replacing the clinical examination for both diagnosing and the follow-up of disease activity. MRI is not recommended for routine use since it is an expensive and inadequate method to show several joints at once [5]. However, the use of US in the field of rheumatology is increasing every day due to its advantages such as being easily accessible, repeatable, inexpensive, and radiation-free. US has an excellent correlation with clinical findings and inflammatory markers and is even more valuable than clinical examination for the detection of subclinical synovitis. Synovitis, tenosynovitis, and even erosions of the bone may be revealed with gray-scale US (GSUS) and Power Doppler US (PDUS) [6,7]. It has been shown that GSUS detects synovitis, and PDUS more precisely distinguishes acute and chronic disease in the synovium [8].

In light of this information, the objective of this study is to evaluate the hand-wrist findings of RA patients using synthetic and a combination of biological and synthetic disease-modifying antirheumatic drugs (DMARDs) in terms of ultrasonographic, clinical, and radiographic data.

## Materials and methods

The clinical study was planned as a cross-sectional study. Thirty-one RA patients followed in the rheumatology outpatient clinic between September 2021 and March 2022 were enrolled in the study. Ethical approval was obtained before the initiation of the study (Local Ethics Committee of Bakirkoy Sadi Konuk Training and Research Hospital, no: 2021/445). Written and informed consent was taken from all patients.

Inclusion criteria for the study were as follows: being diagnosed with RA for more than five years according to the 2010 American College of Rheumatology/European League Against Rheumatism (ACR/EULAR) classification criteria, clinically active or previously proven hand involvement (tenderness, swelling), and had received synthetic (methotrexate, hydroxychloroquine, sulfasalazine, leflunomide)/biological (TNF inhibitors: etanercept, adalimumab, and infliximab) DMARDs for at least six months. Patients with a history of cancer or any hematological abnormality, being pregnant or women in the postpartum period (first six months), trauma history to the hand and wrist, having septic or crystal arthropathy, or a history of previous surgery were excluded from the study.

The patients were divided into two groups according to the order of admission to the rheumatology outpatient clinic. Each patient who did not include the exclusion criteria was enrolled in the study. The patients were divided into two groups according to the treatment they received: synthetic DMARD (n=19) treatment and a combination of synthetic and biological DMARD (n=12) treatment. Age, gender, dominant hand, duration of drug use, duration of complaints, and comorbidities were recorded for each group. Clinical, radiographic, and laboratory findings were evaluated by a single rheumatologist (HH) for all patients. Then, ultrasonographic evaluations of both hands, which lasted for 30 minutes, were performed on the same day by the researchers (CC and YED) who were blinded to clinical and laboratory data.

Disease Activity Score-28 (DAS-28), which includes the CRP level, the number of swollen and tender joints, and the patient's global health assessment, was calculated in terms of disease activation in all of the patients. DAS-28 indicates low disease activity between the scores 2.6 and 3.2, moderate disease activity between the scores 3.2 and 5.1, and high disease activity if the score is greater than 5.1 [9]. The Health Assessment Questionnaire (HAQ) score consisting of 20 questions was calculated for the assessment of the quality of life and general health status of all patients [10].

Ultrasonographic assessment

An ultrasonographic evaluation was performed using MyLab 60 high-resolution 7-12 MHz linear probe US device (Esaote Biomedica, Italy). The wrist was scanned from the dorsal and palmar aspects; fingers were evaluated from the dorsal side in transverse and longitudinal planes. Thirteen joint sites were evaluated, thus 26 joints in both extremities in total. These joints are as follows: the first-fifth metacarpophalangeal (MCP), the first-fifth proximal interphalangeal (PIP), and radiocarpal, midcarpal, and ulnocarpal joints.

All examinations were performed by the investigator with four years of US experience (CC) and repeated by another investigator having two years of US experience (YED). A total of 26 joints were assessed with gray-scale US (GSUS) and Power Doppler US (PDUS). The total synovitis grade score was calculated using the EULAR-OMERACT combined scoring system [11]. This score is obtained by combining the GSUS and PDUS scores [12,13].

In GSUS, synovitis was classified using the semiquantitative scoring method (0-3 scale; Grade 0: no synovial thickening, Grade 1: minimal synovial thickening, Grade 2: swelling of synovial thickening on the line connecting the tops of the periarticular bones, but not extending across the bony diaphysis, and Grade 3: synovial thickening on the line connecting the tops of the periarticular bones and extending to at least one of the bone diaphyses) [14,15]. Synovial vascularization was assessed by PDUS, and synovitis was classified using semiquantitative technical features (0-3 scales; Grade 0: minimal/no vascularity, Grade 1: single vessel signals, Grade 2: combined vessel signals in less than half the synovium area, and Grade 3: vessel signals in more than half the synovium area) [15,16]. In addition, flow was demonstrated in both planes (transverse and sagittal planes). The effusion was defined as hypoechoic or anechoic compressible intraarticular material within the synovial recesses. Synovitis was defined as echogenic incompressible intra-articular tissue within the synovial recesses [17].

Radiological evaluation

Total joint space and total erosion scores of all patients were evaluated with posteroanterior radiographs by an independent radiologist who was blinded for the study. Radiographic damage was determined using the modified Sharp-van der Heijde method [18]. This method measures erosions and joint space narrowing in 32 different joints. Thus, the maximum total erosion score of the hands is 160. The maximum total narrowing score in the hands is 120. For hands only, the Sharp-van der Heijde modified score is 280.

Statistical analysis

SPSS Statistics version 22.0 (IBM Corp. Released 2013. IBM SPSS Statistics for Windows, Version 22.0. Armonk, NY: IBM Corp.) was used for statistical analyses. The distribution of variables was measured using the Kolmogorov-Smirnov test. The Mann-Whitney U test was used for the analysis of quantitative independent data. The chi-square test was used in the analysis of qualitative data. For the evaluation of total synovitis, the US and scores of the more experienced observers were taken into consideration.

## Results

Among 31 patients included in the study. The mean age of the patients was 56.3 ± 9.3 years. When the patients who received a combination of biological and synthetic DMARDs and those who received only synthetic DMARDs were compared in terms of age, sex, dominant hand, duration of drug use, and duration of complaints, there was no significant difference between the two groups. There was no significant difference in comorbidities between the groups (Table 1).

**Table 1:** Comparison of demographic and clinical data for the two groups *: mean±standard deviation, a statistically significant test result (p≤0.05), DMARD: disease-modifying antirheumatic drugs, DM: diabetes mellitus, HT: hypertension

	Synthetic DMARDs	Biological + synthetic DMARDs	p
Age*	57.4+9.6	54.7+8.9	0.447
Gender (%)			0.543
Female	18 (%95)	10 (%83)	
Male	1 (%5)	2 (%17)	
Dominant hand (%)			0.387
Right	19 (%100)	11 (%92)	
Left	-	1 (%8)	
Duration of drug use (months)*	129.4+103.8	127.7+91.6	0.963
Duration of complaints (months)*	139.6+103.3	147.0+102.8	0.847
Comorbidities (%)			
HT	6 (32%)	3 (25%)	1.000
DM	2 (11%)	2 (17%)	0.630
Cardiac diseases	1 (5%)	2 (17%)	0.543
Thyroid diseases	2 (11%)	1 (8%)	1.000

When the groups were compared in terms of ESR, CRP, number of tender and swollen joints, total joint erosion, DAS-28, and HAQ scores, there was no significant difference between the groups. 25-OH vitamin D levels were significantly higher only in the group receiving synthetic DMARDs (p=0.008). The total joint space score was significantly higher in the group receiving a combination of biological and synthetic DMARDs (p=0.047) (Table 2).

**Table 2:** Comparison of laboratory and clinical evaluations for the two groups *: mean±standard deviation, **: median (interquartile range), a statistically significant test result (p≤0.05), DMARDs: disease-modifying antirheumatic drugs, ESR: erythrocyte sedimentation rate, CRP: C-reactive protein, DAS-28: Disease Activity Score-28, HAQ: Health Assessment Questionnaire, GSUS: gray-scale ultrasonography, PDUS: Power Doppler ultrasonography

	Synthetic DMARDs	Biological + synthetic DMARDs	p
ESR*	25.2+18.6	31.9+39.5	0.528
CRP**	3.0 (0.3-30)	6.1 (0.1-146)	0.887
25-OH D vit*	24.9+10.0	14.9+8.4	0.008*
Number of tender joints*	2.6+2.6	3.8+4.0	0.348
Number of swollen joints**	1.0 (0-8)	0.5 (0-7)	0.697
DAS-28*	3.8+1.1	3.8+1.6	0.913
HAQ*	10.8+8.2	9.0+9.1	0.554
Total joint space score**	32 (30-58)	35 (30-72)	0.047*
Total erosion score**	32 (32-68)	33 (32-76)	0.458
Van der Heijde modified score**	64 (62-126)	68 (62-148)	0.083
Total GSUS synovitis score*	16.5+10.0	17.0+9.6	0.909
Total PDUS synovitis score**	0 (0-16)	0 (0-4)	0.296

When the groups were compared in terms of right and left PIP, MCP, and radiocarpal, ulnocarpal, and midcarpal synovitis grade total scores, no significant difference was found between the two groups (Table 3). Interobserver agreement rate, κ value for total synovitis score accuracy is 0.78.

**Table 3:** Synovitis grade total score *: median (interquartile range), a statistically significant test result (p≤0.05), DMARDs: disease-modifying antirheumatic drugs, PIP: proximal interphalangeal, MCP: metacarpophalangeal

		Synthetic DMARDs	Biological + synthetic DMARDs	p
Right PIP*	1.	1.0 (0-2)	0.0 (0-2)	0.399
	2.	0.0 (0-3)	0.5 (0-2)	0.088
	3.	0.0 (0-2)	1.0 (0-2)	0.452
	4.	0.0 (0-2)	0.0 (0-2)	1.000
	5.	0.0 (0-2)	0.0 (0-1)	0.512
Left PIP*	1.	1.0 (0-2)	0.5 (0-2)	0.888
	2.	0.0 (0-2)	0.0 (0-1)	0.730
	3.	0.0 (0-2)	1.0 (0-1)	0.822
	4.	0.0 (0-2)	0.0 (0-3)	0.494
	5.	0.0 (0-2)	0.0 (0-1)	0.566
Right MCP*	1.	1.0 (0-3)	1.0 (0-2)	0.464
	2.	0.0 (0-2)	1.5 (0-3)	0.106
	3.	1.0 (0-2)	1.0 (0-2)	0.191
	4.	1.0 (0-2)	1.0 (0-2)	0.721
	5.	0.0 (0-2)	1.0 (0-2)	0.656
Left MCP*	1.	0.0 (0-2)	0.0 (0-1)	0.239
	2.	1.0 (0-2)	1.0 (0-2)	1.000
	3.	1.0 (0-3)	1.0 (0-2)	0.864
	4.	1.0 (0-3)	0.5 (0-2)	0.868
	5.	1.0 (0-2)	0.0 (0-1)	0.200
Right radiocarpal*		0.0 (0-3)	0.0 (0-2)	0.831
Left radiocarpal*		0.0 (0-2)	0.0 (0-2)	1.000
Right ulnocarpal*		0.0 (0-3)	0.5 (0-2)	0.213
Left ulnocarpal*		0.0 (0-3)	0.0 (0-2)	1.000
Right midcarpal*		0.0 (0-3)	0.5 (0-2)	0.059
Left midcarpal*		0.0 (0-2)	0.0 (0-2)	0.841

## Discussion

In our study, no difference was found in RA patients using synthetic DMARD or a combination of synthetic and biological DMARDs in terms of synovitis evaluated ultrasonographically. Radiographically, the total joint space was significantly narrower, and 25-OH vitamin D levels were significantly lower in the group receiving combination therapy. Radiographic joint erosion was also higher in the group using the combination treatment, but there was no significant difference between the two groups.

According to our study, no difference was detected between the two groups in terms of demographic characteristics, comorbidity status, duration of drug use, and clinical and laboratory disease activation markers. Considering these data, it can be concluded that the two groups are homogeneous in this respect.

For both groups, the total synovitis grade score calculated according to the EULAR-OMERACT combined scoring system did not differ significantly in the ultrasonographic evaluation of bilateral first-fifth PIP, MCP, and radiocarpal, ulnocarpal, and midcarpal joints. In the study conducted by Cruces et al., similar to our study, no significant difference was found between RA patients using synthetic DMARDs or a combination of synthetic and biological DMARDs in terms of subclinical synovitis detected by US [19]. In a study by Saleem et al., gray-scale synovitis scores were found to be higher in the group using a combination of synthetic and biological DMARD treatment compared to the group using only synthetic DMARDs. However, PD synovitis scores were found to be similar between the two groups [20]. Apart from these studies, the radiographic damage degrees between the two groups were also compared in our study, and the total joint space score was found to be significantly higher in the group receiving combination therapy.

In the literature, it has been shown that the progression of joint damage persists despite clinical remission, and this situation is associated with subclinical inflammation demonstrated by imaging methods (MRI-US) [21,22]. According to Harman et al., it takes three months to get an ultrasonographic recovery response in patients under synthetic DMARDs and low-dose corticosteroid treatments. In addition, considering the US findings, Harman et al. suggested that persistent synovitis despite DMARD treatment may lead to radiographic bone erosions in patients having late diagnosis [23]. Similarly, in our study, the time from the onset of symptoms to diagnosis and the bone erosion score were higher in the group using a combination of synthetic and biological DMARDs.

In other studies, it has been shown that there is an improvement in PD signals two to six weeks after the initiation of TNF inhibitor treatment [24,25]. In addition, it has been shown that radiographic deterioration is closely associated with the persistence of the PD signal but not with high baseline PD scores [23,26,27]. This explains why radiographic joint damage progresses, although patients achieve remission according to ACR criteria and DAS-28 scores. In this study, it can be concluded that the delayed diagnosis of the group receiving combination therapy caused the joints to be exposed to long-term subclinical inflammation and thus increased joint damage. These high radiographic damage scores show the importance of ultrasonographic follow-up in routine examinations as well as early diagnosis and treatment. This is because active synovitis is shown on US in approximately one-third of clinically normal-appearing joints. This reflects the superior sensitivity of imaging modalities such as MRI and US in detecting synovial inflammation compared to clinical examination [21].

In this study, 25-OH vitamin D levels were significantly lower in the group receiving combination therapy. A meta-analysis revealed that vitamin D levels were lower in RA patients compared to healthy people, and there was a negative correlation between disease activity and vitamin D levels [28]. In our study, there was no significant difference in disease activity (DAS-28) between the groups; however, vitamin D levels were lower in the group receiving combination treatment.

Our study is important and adds to the literature for detecting the chronic period of hand-wrist findings of RA and revealing the damage that occurs according to the medical treatment used. As a matter of fact, in our study, the total joint space score was found to be significantly higher in the group using a combination of synthetic and biological DMARDs compared to the group receiving synthetic DMARD solely. Although the erosion score was not significant, it was higher in the group using a combination of synthetic and biological DMARDs. Another profit of our study is that it is appropriate for daily practice. In addition to clinical and ultrasonographic comparisons, radiographic alterations were evaluated with a standard method, which is another strength of our study.

The limitation of our study was that not all patients were in clinical remission. However, since there was no significant difference between the groups in terms of disease activity, the outcome of the study was not affected by this condition. Financial evaluations of the treatments were not made in our study. Additionally, the long-term results of the patients were not evaluated. Our results should be supported by studies with higher patient numbers.

## Conclusions

In conclusion, considering the US findings, persistent synovitis despite DMARD treatment may cause radiographic bone erosions. Therefore, it can be concluded that the utilization of imaging systems as an assessment tool to accurately measure synovitis levels in patients with stable disease may provide additional objective information to guide treatment decisions. With the results of this study, we have shown the importance of making immediate treatment protocol changes to suppress inflammation in order to prevent joint damage in the long term. In light of these data, existing RA remission criteria may be modified to optimize the evaluation of disease activity and to select the most appropriate treatment for patients.
